# The impact of birth mode of delivery on childhood asthma and allergic diseases–a sibling study

**DOI:** 10.1111/j.1365-2222.2012.04021.x

**Published:** 2012-08-28

**Authors:** C Almqvist, S Cnattingius, P Lichtenstein, C Lundholm

**Affiliations:** 1 Department of Medical Epidemiology and BiostatisticsStockholm, Sweden; 2 Department of Women's and Children's Health, Astrid Lindgren Children's HospitalStockholm, Sweden; 3 Clinical Epidemiology Unit, Department of Medicine, Karolinska University Hospital and Karolinska InstitutetStockholm, Sweden

**Keywords:** asthma, caesarean section, confounding factors, siblings, twin, vaginal birth

## Abstract

**Background:**

Caesarean section (CS) has been reported to increase the risk of asthma in offspring. This may be due to that infants delivered by CS are unexposed to vaginal flora, according to the ‘hygiene hypothesis’.

**Objective:**

Our aim was to investigate if CS increases risk of childhood asthma, and if the risk increase remains after adjustment for familial confounding using sibling design.

**Methods:**

A register-based cohort study with 87 500 Swedish sibling pairs was undertaken. Asthma outcome variables were collected from national health registers as diagnosis or asthma medication (ICD-10 J45-J46; ATC code R03) during the 10th or 13th year of life (year of follow-up). Mode of delivery and confounders were retrieved from the Medical Birth Register. The data were analysed both as a cohort and with sibling control analysis which adjusts for unmeasured familial confounding.

**Results:**

In the cohort analyses, there was an increased risk of asthma medication and asthma diagnosis during year of follow-up in children born with CS (adjusted ORs, 95% CI 1.13, 1.04–1.24 and 1.10, 1.03–1.18 respectively). When separating between emergency and elective CS the effect on asthma medication remained for emergency CS, but not for elective CS, while both groups had significant effects on asthma diagnosis compared with vaginal delivery. In sibling control analyses, the effect of elective CS on asthma disappeared, while similar but non-significant ORs of medication were obtained for emergency CS.

**Conclusions and Clinical Relevance:**

An increased risk of asthma medication in the group born by emergency CS, but not elective, suggests that there is no causal effect due to vaginal microflora. A more probable explanation should be sought in the indications for emergency CS.

## Introduction

The prevalence of childhood asthma and allergic disease increased up to recently in many nations around the world, which implies that a substantial proportion of the risk of having the disease is attributable to environmental factors 1,2. Pre- and perinatal etiological factors are of great importance, and recent findings have shown that fetal growth affects the susceptibility to asthma 3 and eczema 4. There has also been a large interest in the impact of mode of delivery on the development of asthma. Recent meta-analyses reported a 20% increase in the subsequent risk of asthma in children who had been delivered by Caesarean section (CS) 5,6 and a moderate risk increase for allergic rhinitis and asthma 7. However, results are conflicting; some recent articles have reported children born with CS to have an increased risk of asthma 8–12 whereas others found no such associations 11–14.

The rationale why CS could increase the risk of asthma has been referred to a modified intestinal microflora in those unexposed to the vaginal flora, according to the ‘hygiene hypothesis’ 15. It has also been recently shown by Schlintzig et al. that DNA-methylation is higher in infants delivered by CS than in vaginally delivered infants 16. However, as CS is commonly performed because of concern of maternal and fetal health, a possible association between CS and risk of asthma in offspring may be explained by the underlying *indications* for CS 17,18. These could be related to choice of elective CS (anxious mother, anthropometric measures or obstetric history), emergency situations (prematurity, prolonged parturition or fetal asphyxia) or subsequent diagnoses including early respiratory stress. The association between mode of delivery and asthma and its possible mechanisms can be studied in a dataset where vaginal delivery (VD) is separate from vaginal instrumental delivery, CS can be subdivided into emergency CS and elective CS and unmeasured familial confounding can be taken into consideration using sibling controls. Sibling studies provide an excellent opportunity to study the association between CS and asthma, controlling for shared (familial) environmental and genetic factors. Siblings share half of their genes, some intrauterine exposures, maternal factors and early environment including socio-economic factors, diet and lifestyle. In addition, siblings may be discordant regarding mode of delivery. If associations seen in a cohort of siblings remain in sibling control analyses, then factors specific to each individual (such as exposure to vaginal flora or the indication for mode of delivery) are involved in the underlying causal pathways. Conversely, if the relationships disappear or substantially diminish in sibling control analyses, then factors common to the siblings (such as maternal factors) may be involved.

The aim of this study was to examine the impact of mode of delivery on the prevalence of asthma in childhood and adolescence and the causal mechanism behind a possible link, including sibling analyses to control for unmeasured familial confounding. We used a sibling population with data on mode of delivery and other perinatal exposures as well as asthma medication and specialist care diagnoses from the Swedish national health registers.

## Material and methods

### Study design and population

A population-based follow-up study based on the Swedish national registers held by the Swedish National Board of Health and Welfare and Statistics Sweden was conducted. The universal use of the Personal Identity Number (PIN), a unique identifier for each resident, enables unambiguous linkage between the registers 19. All children born in Sweden from June 1993 to June 1999 were identified through the Medical Birth Register, which contains maternal and child characteristics data on > 98% of all births in Sweden. Information on mother's country of birth and family relationships was retrieved from the Multi-Generation Register, which contains links between parent and child, and vital statistics were retrieved from the Total Population Register. The cohort was limited to the first two siblings of mothers who had given birth to those two children within the allocated time frame. There were 93 740 sibling pairs born June 1993–June 1999. After exclusion of pairs with at least one child coming from a multiple birth (*n* = 1784), pairs with one sibling deceased before end of follow-up (*n* = 776), pairs with at least one sibling without record in the Medical Birth Register (*n* = 2300) and pairs with at least one sibling having contradictory or unclear information on mode of delivery in the Medical Birth Register (*n* = 1325, mainly CS which could not be classified), 87 555 sibling pairs (175 110 children) remained in the cohort.

The cohort was linked to the Prescribed Drug Register (PDR) which contains all prescribed drugs dispensed since July 1, 2005 and the National Patient Register (NPR) which covers all in-patient care in Sweden from 1987 and 75% of all outpatient visits since 2001.

### Variables

#### Birth outcomes

Information on child (birth weight, gestational age [GA], gender, Apgar score and maternal characteristics (age, parity, smoking, family situation, height and weight) was obtained from the Medical Birth Register. In Sweden, early second trimester ultrasonography to estimate GA is routinely offered since 1990, and 95% of women accept this offer, otherwise the date of the last menstrual period is used 20. Apgar score at 5 min was categorized as 0–3, 4–6 and 7–10. Maternal age and parity were recorded at delivery. Mother's smoking habits (0, 1–9 or ≥ 10 cigarettes per day) and family situation (cohabits with child's father yes/no) were registered at first visit to the antenatal-care clinic in week 8–12, along with height and weight, used for calculation of body mass index, kg/m^2^ [BMI]. Mode of delivery was defined as either VD or CS. Elective CS was defined as CS before onset of labour and emergency as CS after onset of labour. Instrumental delivery (vacuum extraction or forceps) was also noted.

Information on type of siblingship; full or half (same mother, different father) and birth order (first or second) was obtained through linkage with the Multi-Generation register.

#### Asthma medication and diagnosis

Dispensed asthma medications were retrieved from the PDR. In our analyses we had two asthma outcome variables based on medication; (1) any asthma medication (ATC codes R03CC, R03AC, R03BA, R03AK, R03DC) and (2) any asthma medication except oral β_2_-agonists, dispensed at least twice during the year of follow up; during the 13th year of life for children born June 1993–May 1996 and during the 10th year of life for those born June 1996–June 1999. A third outcome variable was based on diagnoses of asthma in the NPR (ICD-9 codes 493 and ICD-10 codes J45, J46), during the year of follow up.

### Statistical analyses

The statistical analyses were performed in two steps. First, the associations between asthma medication and mode of delivery were analysed treating the study group as a cohort, with asthma medication and asthma diagnosis as outcome variables using generalized estimating equations (with the logit link) to account for the correlation within sibling pairs. Odds ratios and 95% confidence intervals are reported both as crude and adjusted for the child characteristics gender, birth order, birth weight, GA and Apgar score at 5 min as well as the maternal characteristics age at delivery, smoking during pregnancy, mother living with father of the child, mother's birth country and mother's BMI.

Secondly, to adjust for familial environmental confounding and partly for genetic factors, sibling analyses were performed, using conditional logistic regression within siblings who were discordant for both mode of delivery (i.e. discordant for the four level mode of delivery variable) and the outcome variable. The paired analyses were adjusted for the same variables as the cohort analyses, except mother's birth country, which is the same for both siblings. Furthermore, the conditional analysis produces estimates that are adjusted for all the unmeasured familial factors that siblings have in common, but which are difficult to measure, e.g. parental factors, home environment, life style and to some extent genes. SAS version 9.2 was used for all analyses.

Permission for the study was obtained from the Regional Ethical Review board in Stockholm, Sweden.

## Results

In the main sibling population, 9.4% were delivered with CS, of which 5.4% were emergency and 4.2% elective caesareans. There were 7.6% vaginal instrumental deliveries (7.1% vacuum extractions [VE] and 0.5% forceps). In 20 493 sibling pairs, siblings were born with different modes of delivery. This group of siblings were thus discordant for mode of delivery, and used in the sibling analyses below. In total, 7.0% of the children had any asthma medication (6.8% of those delivered with VD, 7.9% if elective CS and 8.2% if born with emergency CS) and 1.6% of the children had an asthma diagnosis (1.5% of those delivered with VD, 2.0% if elective CS and 2.1% if born with emergency CS).

Table 1 shows child characteristics at birth, subsequent asthma medication and asthma diagnoses, siblingship and maternal characteristics for the full cohort of children and the group discordant for mode of delivery. In the full cohort, both elective and emergency CS were more common in children with low birth weight, short GA and low Apgar scores. The CS group had slightly more dispensed asthma medication and diagnoses of asthma. Both elective and emergency CS rates increased with maternal age and BMI. In the group of siblings with discordant mode of delivery, the pattern was almost identical.

**Table 1:** Study population characteristics by mode of delivery in a cohort of 175 110 Swedish children in sibling pairs, born June 1993–June 1999, and in a subset of the cohort with 40 986 siblings in pairs discordant regarding mode of delivery

	Mode of delivery									
Full cohort					Children in discordant pairs				
*N*	Vaginal	Vaginal with instrument	Caesarean, emergency	Caesarean, elective	*N*	Vaginal	Vaginal with instrument	Caesarean, emergency	Caesarean, elective
*N*	175 110	83.0	7.6	5.2	4.2	40 986	40.5	29.5	16.4	13.7
Child's characteristics										
Birth weight (grams)										
≤ 2999	20 898	76.4	6.4	7.4	9.8	5700	28.9	21.7	22.2	27.2
3000–3499	55 556	84.8	7.1	3.9	4.2	11 730	41.2	30.3	14.0	14.6
3500–3999	63 967	84.7	7.9	4.5	2.9	14 370	43.5	31.7	14.5	10.3
4000–4499	27 872	82.2	8.8	6.4	2.7	7068	43.4	31.3	16.8	8.5
≥ 4500	6195	75.4	8.8	11.2	4.6	1928	38.9	24.3	25.4	11.5
Missing	622	75.6	8.8	6.8	8.8	190	35.8	24.2	16.8	23.2
Gestational age (weeks)										
≤ 34	2371	54.7	2.9	12.2	30.2	1047	16.2	6.1	23.4	54.3
35–36	4903	76.9	5.2	8.2	9.6	1305	29.9	17.5	25.0	27.7
37–38	29 671	77.3	4.8	4.4	13.5	7566	30.3	16.9	13.0	39.9
39–40	93 562	87.3	7.1	3.9	1.7	19 023	48.2	31.5	13.6	6.8
41–42	43 428	80.2	11.1	7.7	1.1	11 623	38.6	37.4	20.9	3.1
≥ 43	1048	64.9	15.7	17.6	1.8	384	21.6	40.6	34.4	3.4
Missing	127	78.7	6.3	7.9	7.1	38	44.7	18.4	18.4	18.4
Gender										
Male	89 930	81.7	8.6	5.7	4.1	21 804	38.2	31.8	17.2	12.9
female	85 180	84.3	6.7	4.8	4.3	19 182	43.2	26.8	15.4	14.6
APGAR score at 5 min										
≤ 3	399	65.2	12.0	14.3	8.5	150	30.7	26.7	26.0	16.7
4–7	2456	46.6	24.7	20.4	8.3	1267	15.5	41.6	30.5	12.4
8–10	170 914	83.5	7.4	5.0	4.1	39 265	41.3	29.2	15.8	13.7
Missing	1341	83.5	4.9	6.0	5.6	304	45.4	19.7	18.1	16.8
Any asthma medication										
No	162 850	83.1	7.6	5.2	4.1	37 922	40.7	29.4	16.2	13.7
Yes	12 260	81.0	8.2	6.1	4.7	3064	38.0	30.1	17.9	14.1
Asthma medication other than oral β_2_-agonists at least twice in 12 months										
No	167 483	83.0	7.6	5.2	4.1	39 051	40.6	29.5	16.4	13.7
Yes	7627	80.9	8.1	6.1	5.2	1935	38.0	29.2	18.4	14.4
Asthma diagnosis in specialist care during 12 months followup†										
No	172 324	83.0	7.6	5.2	4.2	40 273	40.7	29.4	16.3	13.7
Yes	2786	78.9	9.0	6.9	5.2	713	31.8	33.0	20.6	14.6
Type of siblingship										
Full	171 016	82.9	7.7	5.2	4.2	40 136	40.5	29.5	16.3	13.7
Half ‡	4094	84.4	6.6	5.1	3.9	850	42.5	27.7	16.9	12.9
Birth order										
First	87 555	77.0	12.6	7.1	3.2	20 493	15.0	50.8	24.5	9.7
Second	87 555	88.9	2.7	3.3	5.1	20 493	66.0	8.1	8.2	17.7
Mother's characteristics										
Mother's age at delivery (years)										
≤ 19	3985	87.1	6.8	3.9	2.3	622	25.6	41.8	21.2	11.4
20–24	39 727	85.2	7.4	4.6	2.8	7618	32.8	36.1	19.3	11.8
25–29	73 309	83.7	7.8	5.0	3.6	16 327	39.5	31.5	16.6	12.4
30–34	46 125	81.5	7.6	5.7	5.3	12 375	45.3	24.9	14.6	15.2
≥ 35	11 964	75.8	8.2	7.2	8.9	4044	46.8	20.4	14.5	18.2
Smoking during pregnancy (cigarettes per day)										
0	148 546	83.2	7.7	5.1	4.1	34 608	40.9	29.6	16.1	13.4
1–9	13 059	81.2	8.6	5.7	4.5	3133	35.1	32.4	18.0	14.5
≥ 10	5447	82.6	7.1	5.9	4.4	1265	37.5	28.0	20.4	14.2
Missing	8058	83.0	6.4	5.3	5.4	1980	43.8	22.8	16.0	17.4
Cohabits with child's father										
Yes	160 593	83.1	7.6	5.2	4.2	37 633	40.9	29.3	16.1	13.7
No	4635	82.4	9.2	5.2	3.3	999	31.2	38.4	18.9	11.5
Missing	9882	82.0	7.6	5.8	4.7	2354	38.1	28.2	18.6	15.2
Country of birth										
Sweden	119 968	81.8	8.9	5.6	3.2	27 822	34.2	35.3	18.4	12.1
Nordic country	3299	81.0	8.1	5.9	3.7	840	40.7	27.5	18.0	13.8
Other	51 654	85.9	4.7	4.3	5.0	12 279	54.8	16.4	11.6	17.2
Missing	189	85.7	6.9	4.2	5.1	45	48.9	26.7	11.1	13.3
BMI (kg/m^2^)										
< 18.5	4606	86.1	7.3	3.3	3.3	898	40.4	33.2	13.8	12.6
18.5–24.9	101 676	84.1	7.7	4.5	3.7	22 416	40.0	31.7	15.6	12.8
25–29.9	31 414	81.2	7.4	6.5	4.9	8227	43.0	25.1	17.4	14.6
≥ 30	9850	77.7	7.2	8.6	6.5	2896	41.5	22.1	19.3	17.1
Missing	27 564	82.1	8.0	5.4	4.5	6549	38.7	30.2	16.9	14.3

† 13th year of life for children born June 1993–May 1996, 10th year of life for children born June 1996–June 1999.

‡ Same mother, different fathers.

Table 2 presents a cohort analysis on the associations between mode of delivery and the outcomes (asthma medication and asthma diagnoses). When asthma was defined as having taken asthma medication at least twice, there was an increased risk of asthma in children born with CS (OR 1.20, 95% CI 1.11–1.29) in the crude analysis, which remained after adjustment for potential confounders (OR 1.13, 95% CI 1.04–1.24). For asthma diagnosis during the follow-up year, crude and adjusted ORs were also increased (ORs 1.32, 95% CI 1.18–1.48 and 1.20, 95% CI 1.05–1.37 respectively).

**Table 2:** Odds ratios and 95% confidence interval of association between mode of delivery and (i) filled prescriptions of asthma medication at age 9–12 years§ and (ii) asthma diagnosis, in a cohort of children born in Sweden in June 1993–June 1999

	Mode of delivery	Model A* (*n* = 175 110)	Model B† *(n* = 174 381)	Model C‡ (*n* = 139 610)
Any asthma medication	Vaginal	Ref	Ref	Ref
Caesarean	1.18 (1.11–1.25)	1.13 (1.06–1.20)	1.10 (1.03–1.18)
β2-agonists, corticosteroids or montelucast, at least twice	Vaginal	Ref	Ref	Ref
Caesarean	1.20 (1.11–1.29)	1.15 (1.06–1.24)	1.13 (1.04–1.24)
Asthma diagnosis during follow up year‡	Vaginal	Ref	Ref	Ref
Caesarean	1.32 (1.18–1.48)	1.23 (1.10–1.39)	1.20 (1.05–1.37)

* Adjusted for gender.

† Adjusted for gender, birth weight and gestational age.

‡ Adjusted for gender, birth weight, gestational age, birth order, APGAR score, hypoxia/asphyxia, mother's age, smoking during pregnancy, mother living with father of the child, mother's birth country and mother's BMI.

§ 13th year of life for children born June 1993–May 1996, 10th year of life for children born June 1996–June 1999.

In Table 3, the CS group was stratified into emergency and elective CS, to study whether the CS per se or the indicators for CS where important for asthma risk in the whole cohort. The estimates remained stable for emergency CS, but were slightly lower and generally not statistically significant for elective CS. Also, we compared VS with instrumental VD and found no difference.

**Table 3:** Odds ratios and 95% confidence interval of association between mode of delivery and (i) filled prescriptions of asthma medication at age 9–12 years¶ and (ii) asthma diagnosis, in a cohort of children born in Sweden in June 1993–June 1999

	Mode of delivery	Model A* (*n* = 175 110)	Model B† (*n* = 174 381)	Model C‡ (*n* = 139 610)
Any asthma medication	Vaginal	Ref	Ref	Ref
Vaginal, instrumental	1.08 (1.01–1.16)	1.09 (1.02–1.17)	1.03 (0.95–1.12)
Caesarean, emergency	1.19 (1.10–1.29)	1.17 (1.08–1.27)	1.14 (1.04–1.25)
Caesarean, elective	1.18 (1.08–1.29)	1.09 (0.99–1.19)	1.06 (0.95–1.18)
β2-Agonists, corticosteroids or montelucast, at least twice	Vaginal	Ref	Ref	Ref
Vaginal, instrumental	1.05 (0.97–1.14)	1.06 (0.98–1.16)	1.02 (0.92–1.13)
Caesarean, emergency	1.18 (1.07–1.30)	1.17 (1.06–1.29)	1.16 (1.03–1.29)
Caesarean, elective	1.24 (1.11–1.38)	1.13 (1.01–1.27)	1.10 (0.97–1.26)
Asthma diagnosis during follow up year¶	Vaginal	Ref	Ref	Ref
Vaginal, instrumental	1.20 (1.05–1.37)	1.21 (1.06–1.38)	1.05 (0.91–1.23)
Caesarean, emergency	1.35 (1.16–1.57)	1.32 (1.14–1.54)	1.18 (1.00–1.34)
Caesarean, elective	1.33 (1.12–1.58)	1.17 (0.98–1.40)	1.25 (1.02–1.52)

† Adjusted for gender.

† Adjusted for gender, birth weight and gestational age.

† Adjusted for gender, birth weight, gestational age, birth order, APGAR score, hypoxia/asphyxia, mother's age, smoking during pregnancy, mother living with father of the child, mother's birth country and mother's BMI.

† 13th year of life for children born June 1993–May 1996, 10th year of life for children born June 1996–June 1999.

A comparison between VD born children with VD and CS siblings and CS born children with VD and CS siblings showed very similar prevalence between all groups except CS born children with CS siblings, who had statistically significantly higher odds of diagnosis during follow-up compared with the other groups (data not shown). This pattern was not as apparent for the other outcome variables.

Table 4 shows results from the sibling control analysis, i.e. the sibling pairs with discordant mode of delivery and asthma status, which by design adjusts for shared familial factors. There was a borderline increased risk of asthma medication at least twice in children born with emergency CS (adjusted OR 1.24, 95% CI 0.99–1.60) but not significant for elective CS (OR 0.82, 95% CI 0.64–1.09). Similarly, there was a borderline increased risk of any asthma medication in children born with emergency CS (OR 1.19, 95% CI 0.98–1.44). For asthma diagnosis during the year of follow-up, ORs were equally non-significant after adjustment. The difference in effect between emergency vs. elective CS was statistically significant, *P* = 0.0033.

**Table 4:** Odds ratios and 95% confidence interval of association between mode of delivery and (i) filled prescriptions of asthma medication at age 9 or 12 years¶ and (ii) asthma diagnosis, in sibling pairs discordant on mode of delivery within a cohort of children born in Sweden in June 1993–June 1999

	Mode of delivery	Model A*	Model B†	Model C‡
Any asthma medication	Vaginal	Ref	Ref	Ref
Vaginal, instrumental	1.02 (0.92–1.13)	1.02 (0.91–1.13)	0.94 (0.82–1.08)
Caesarean, emergency	1.23 (1.06–1.42)	1.19 (1.02–1.39)	1.19 (0.98–1.44)
Caesarean, elective	1.05 (0.89–1.23)	0.91 (0.77–1.08)	0.85 (0.68–1.05)
β2-Agonists, corticosteroids or montelucast, at least twice	Vaginal	Ref	Ref	Ref
Vaginal, instrumental	0.95 (0.83–1.08)	0.95 (0.83–1.08)	0.89 (0.64–1.09)
Caesarean, emergency	1.28 (1.06–1.55)	1.23 (1.02–1.49)	1.24 (0.99–1.60)
Caesarean, elective	1.07 (0.87–1.30)	0.92 (0.74–1.13)	0.82 (0.64–1.09)
Asthma diagnosis during follow up year¶	Vaginal	Ref	Ref	Ref
Vaginal, instrumental	1.29 (1.04–1.61)	1.23 (0.99–1.54)	0.85 (0.64–1.13)
Caesarean, emergency	1.81 (1.31–2.49)	1.65 (1.19–2.30)	1.29 (0.84–1.99)
Caesarean, elective	1.10 (0.79–1.51)	0.90 (0.63–1.27)	0.65 (0.42–1.02)

† Adjusted for gender.

† Adjusted for gender, birth weight and gestational age.

† Adjusted for gender, birth weight, gestational age, birth order, APGAR score, mother's age, smoking during pregnancy, mother living with father of the child and mother's BMI.

† 13th year of life for children born June 1993–May 1996, 10th year of life for children born June 1996–June 1999.

## Discussion

In a cohort analysis of 87 555 sibling pairs born 1993–1999, we found an increased risk of asthma in children born with CS. We also found that emergency CS, but not elective CS, was associated with increased risk of asthma, even when we had the best possible controls and adjusted for unmeasured familial confounding in the sibling analyses. Thus our results, based on the largest population-based register linkage study on mode of delivery and asthma to date, support the hypothesis that CS *per se* does not increase the risk of subsequent asthma, but could be explained by the reason for CS, such as factors related to maternal or foeto-infant health.

Previous studies have shown an increased risk of asthma with CS 8–10,21,22. In some 8 but not all 9,10,21,22 studies, risks have been higher in children delivered with emergency CS than in children delivered by elective CS, which was also confirmed in our study. Also studies without any overall positive association between CS and asthma have shown differences in effect between elective and emergency CS, or seen a difference in effect for those born premature or with low birth weight 11,13.

The novelty of this study is the sibling control analysis. Using sibling pairs discordant for mode of delivery makes it possible to adjust for unmeasured familial factors, such as maternal characteristics, family environment and, to some extent, genes, i.e. factors that are shared between siblings. When using sibling controls, our results showed a borderline association between emergency CS and asthma, a non-significant association with point estimates below one between elective CS and asthma and a statistically significant difference in effect between emergency and elective CS. This novel data implies that the association observed between elective CS and asthma in the cohort analyses is not likely to be explained by exposure to vaginal microflora or epigenetic changes. Håkansson et al. also used the Swedish Medical Birth Register linked to asthma diagnoses in the NPR 22. Although they excluded e.g. children with low Apgar scores, some neonatal diagnoses and low or high birth weights, their crude estimates were fairly similar to ours regarding registered asthma diagnoses. They also made a comparison between VD born children with VD and CS siblings (but not CS emergency and CS elective), concluding no difference between the groups. We have extended the analyses further, included data from the PDR, and adjusted for familial confounding with conditional analyses within sibling pairs discordant for mode of delivery. This allowed us to identify a borderline significance for the effect of emergency CS on asthma.

In high risk pregnancies, there are often maternal or fetal indications for CS 17 and higher DNA-methylation have been identified in infants delivered by CS than infants VD born 16. Findings on the effect of CS on asthma and allergic disease have also been proposed to be mediated by the infant's lack of early exposure to vaginal microflora, related to the hygiene hypothesis 9 If so, there would also be a difference in the effect between elective CS and VD, which was not the case in our analyses. There was also no effect of instrumental VD on the risk of asthma in the sibling analyses. Instrumental deliveries are usually undertaken at times of prolonged parturition, tired mother or sick child. An increased risk for asthma would therefore be suspected in these children, however, it has also been speculated that these children are protected by exposure to vaginal microflora and should therefore have a low asthma risk. In our dataset, this is not a likely explanation as we did not see any difference in effect in the sibling analyses.

Strengths of our study include (1) a population-based longitudinal register based study design in a unified health care environment with recording in medical registers of all 9–12 year olds in Sweden; (2) prospectively collected information on mode of delivery, maternal background factors and perinatal characteristics, which preclude recall bias; (3) ascertainment of asthma by applying predetermined asthma criteria to the NPR (covers inpatient and outpatient visits but not visits to general practitioners) and PDR (full coverage of all dispensed asthma medication); and (4) a novel sibling design with identification of sibling pairs discordant for mode of delivery, which adjusts for maternal characteristics and shared unmeasured family environment including socio-economic factors, diet and lifestyle. Within sibling analyses build on similar concept as the co-twin analysis, which is commonly used to adjust for unmeasured familial factors including maternal characteristics, family environment and, to some extent, genes. The matched-sibling design has a number of advantages in controlling for family factors, which are difficult to measure and control for in conventional cohort designs. In addition, we had reasonable statistical power.

There are also inherent limitations in register-based studies with residual confounding due to factors not recorded in the registers. We lack information on genetic factors, many pregnancy associated factors (e.g. maternal diet and physical activity during pregnancy) and other factors related to both mode of delivery and the likelihood of obtaining asthma medication or an asthma diagnosis (e.g. parental behavioural factors related to health care utilization). Importantly, the sibling control analysis control for familial (shared genetic and environmental) factors by design, and therefore reduce the risk of residual confounding. There are limitations to the sibling design that should be mentioned. The strict control for shared family factors limits the analyses to a small subset of the population. Only sibling pairs discordant for mode of delivery as well as asthma outcomes contribute to the estimate in these analyses. In the present study, we had 20 493 sibling pairs which were discordant for mode of delivery, but only 1005 of these pairs were also discordant for any asthma medication and 240 pairs discordant for asthma diagnosis. We find it unlikely that the estimated effect of mode of delivery on asthma is different in the cohort and sibling analyses, as each delivery is individually assessed and observations in both analyses were retrieved from the same study population. Misclassification or underreporting of asthma outcomes may be related to socio-economic factors, which were controlled for (by design) in the sibling analyses. We have no reason to believe that misclassification or underreporting of asthma outcomes differ by mode of delivery.

Future studies based on larger datasets through linkage with several national registers and including sibling controls may have additional power to analyse the association between mode of delivery and asthma. Collection of objective markers of lung function and measures of allergic disease in siblings discordant for CS would also provide important data, as would information on maternal choice of delivery.

In conclusion, our results, based on the first sibling control analysis on mode of delivery and asthma, suggest that the association between CS and risk of asthma are not explained by exposure to the vaginal microflora or early epigenetic changes, but are subject to the underlying indications for CS.
